# Cancer survivors’ views on digital support for smoking cessation and alcohol moderation: a survey and qualitative study

**DOI:** 10.1186/s12889-021-11785-7

**Published:** 2021-09-27

**Authors:** Ajla Mujcic, Matthijs Blankers, Dilara Yildirim, Brigitte Boon, Rutger Engels

**Affiliations:** 1 Trimbos Institute, The Netherlands National Institute of Mental Health and Addiction, Utrecht, The Netherlands; 2 Erasmus School of Social and Behavioural Sciences, Erasmus University Rotterdam, Rotterdam, The Netherlands; 3 Department of Research, Arkin Mental Health Care, Amsterdam, The Netherlands; 4 Department of Psychiatry, Amsterdam UMC, Location AMC, University of Amsterdam, Amsterdam, The Netherlands; 5 Department Adult Mental Health, Mental Health Care Organization GGZ Delfland, Location Schiedam, Schiedam, The Netherlands; 6 Academy het Dorp, Arnhem, The Netherlands; 7 Siza, Arnhem, The Netherlands; 8 Tranzo, Tilburg University, Tilburg, The Netherlands

**Keywords:** Cancer survivors, Qualitative study, Smoking, Tobacco, Alcohol use, eHealth, Digital health

## Abstract

**Background:**

Digital interventions may provide low-threshold support for smoking cessation (SC) and alcohol moderation (AM) to the growing population of cancer survivors. The objective was to explore preconditions of successful AM and SC digital interventions for cancer survivors.

**Methods:**

Using a multi-method approach we conducted a survey (*n* = 240), a qualitative study consisting of four focus groups (*n* = 15) and semi-structured interviews with Dutch cancer survivors (*n* = 8). To help interpretation of our results we interviewed experts in the field of eHealth and cancer survivors (*n* = 6) and we organized an expert meeting (*n* = 7). Qualitative data were analysed using the Framework approach and were double-coded by two coders.

**Results:**

Survey results show the majority of drinkers had not previously considered AM (*n* = 158, 84.9%), often because they deemed their alcohol use to be non-problematic. All current smokers in the survey had considered SC before. In focus groups and interviews it became clear that SC efforts did not always stem from their own willingness to quit smoking, but originated from a wish to please their social environment. Main themes to be addressed in digital SC and AM that emerged from the interviews and focus groups, centred on the different ways of identification as cancer survivors, need for autonomy, differential beliefs about SC and AM, and the importance of a positive, non-patronizing tone-of-voice. Several specific preferences for digital interventions were formulated, although some cancer survivors prefer no support or face-to-face contact.

**Conclusions:**

Cancer survivors are a diverse group with diverse preferences for AM and SC support. Digital AM and SC interventions for cancer survivors are perceived to be of value by some, especially when they incorporate a positive, non-judgemental and non-patronizing tone-of-voice, address concerns specifically relevant to cancer survivors, offer possibilities for personalization, and emphasize autonomy throughout. To encourage AM specifically, problem recognition and awareness of the health benefits of AM should be improved.

**Supplementary Information:**

The online version contains supplementary material available at 10.1186/s12889-021-11785-7.

## Background

Alcohol and tobacco use are among the main behavioural risk factors for cancer [1], but also important risk factors for second cancers and iatrogenic effects of cancer treatment [2, 3]. Yet, rates of smoking and alcohol use among cancer survivors are high. Cancer survivors are defined as people diagnosed with cancer at some point in their lives, irrespective of treatment phase. Currently, there seem to be few effective interventions for alcohol moderation (AM) and smoking cessation (SC) in cancer survivors [4, 5].

A recent meta-analysis of 21 SC interventions for cancer survivors found that SC interventions are not more effective than control groups (d = 0.030, 95%CI − 0.042 to 0.101) (Sheeran, 2019). A meta-analysis on distance-based SC and AM interventions (ie., telephone, print or web-based) found distance-based SC interventions to be more effective than control group interventions (10 studies, OR = 1.56, 95% CI, 1.13–2.15, *P* = .007) [4], but found no evidence for the effectiveness of distance-based AM interventions in three studies, (SMD = 0.12; 95% CI, − 0.08 to 0.31, *P* = .24).

Digital interventions might provide a low threshold for seeking help and reach a large population. They have shown their effectiveness in the general population [6, 7]. To inform the development of two digital interventions for SC and AM we sought to explore the factors contributing to the use of digital support by cancer survivors.

Several previous qualitative studies have described views of cancer survivors and healthcare professionals on smoking [8], SC [9], lifestyle behaviour change [10, 11] and online self-management (e.g., [12]). Recurring themes are (the importance of) enjoying life, being open to discussion of SC with a healthcare professional, and scepticism of benefits of SC (after cancer). Only one study described bladder cancer survivors’ experiences with alcohol cessation, in a peri-operative setting [13], and reported that cancer treatment can function as a teachable moment, an opportunity for positive lifestyle behaviour change, in that way possibly encouraging SC and AM. However, the return to daily life and accompanying shift of focus to day-to-day life increased the risk of relapse. One French study has described health professionals’ views on AM and SC in head and neck cancer survivors [14]. To the best of our knowledge, no study yet has looked specifically into views on digital interventions for SC and AM for cancer survivors.

In this study, we aimed to explore the preconditions for successful digital alcohol and tobacco interventions for cancer survivors. These findings were collected and used to inform the development of digital interventions MyCourse – Quit Smoking and MyCourse – Moderate Drinking, both specifically designed for cancer survivors [15]. This study explored the following research questions:

1. What are cancer survivors’ views on SC and AM?
2. What are cancer survivors’ preferences regarding digital support for SC and AM?
3. What are experts’ recommendations for successful digital AM and SC support for cancer survivors?

## Methods

### Design

To answer our research questions, we employed a multi-method approach. We conducted a survey, focus groups and semi-structured interviews with Dutch cancer survivors. We started with a survey to assess preferences for (digital) support in SC and AM, the survey results subsequently informed the topics that were discussed further during the focus groups. The interviews provided more insight into (altered) interests and concerns after the cancer diagnosis and views on (digital) support. In addition, to better understand preconditions for successful digital SC and AM interventions for cancer survivors, we consulted experts in the field of lifestyle behaviour change, cancer survivorship, design and implementation of digital interventions, SC and AM. Experts were consulted in semi-structured interviews and during an expert meeting to discuss the main findings. The study was performed in accordance with the Consolidated criteria for reporting qualitative research (COREQ) guidelines.

### Recruitment

Cancer survivors for the survey were recruited on the largest Dutch online cancer survivor platform; Kanker.nl. Adult members who - based on their account information - were diagnosed with cancer at some point in their lives, excluding those in palliative phase, and who had agreed to be contacted for participation in scientific studies, received an invitation to the online survey (*n* = 635). Interviewed cancer survivors were a convenience sample recruited using social media and snowball sampling. They were either smokers, former smokers or currently using alcohol. Former smokers were invited because they could share their insights into what helped them quit smoking. Focus group participants were respondents of the survey, who had given consent to be contacted for future studies. They were invited via email and filled out a short questionnaire to indicate their availability. All respondents were contacted and the most suitable dates were selected. Interviewed experts were purposively sampled researchers with experience in development and implementation of digital support for cancer survivors (*n* = 4) and healthcare professionals experienced in supporting SC and AM (*n* = 2). For the expert meeting, we invited researchers with expertise in lifestyle behaviour change, AM and SC, eHealth development, and implementation (*n* = 7).

### Data collection

#### Survey

The survey was conducted during a period of 2 weeks in January 2016. Members of the cancer platform received an online invitation and a one-time online reminder. The online survey focused on alcohol and tobacco use, help-seeking for lifestyle behaviour change and preferences for digital support in cancer survivors. See Additional file 1 for survey questions. It contained both closed and open-ended questions. Alcohol use was assessed using the questions “Do you (sometimes) drink alcohol or do you not drink alcohol at all?”, “How often do you drink alcohol?” and “How much do you usually drink on a drinking day?”, measured in standard units corresponding to 10 g of ethanol per unit. Tobacco use was assessed by the questions “Do you (sometimes) smoke or do you not smoke at all?” and “How many cigarettes do you smoke per day?”. Online informed consent was provided before the start of the questionnaire. The survey lasted approximately 5 min. Survey respondents received no reimbursement for their participation.

#### Interviews

Semi-structured face-to-face interviews were conducted with 8 cancer survivors in April and May of 2016 during the development of the MyCourse interventions. The interviews focused on themes that arose from survey results and the first two focus groups. Specifically, they centred on (altered) interests and concerns after the cancer diagnosis, views on AM and SC and the use of technology in quitting smoking or moderating alcohol use. Written informed consent was obtained from the cancer survivors. Interviews lasted between 30 and 60 min and were conducted at the participants’ home or other location of their choosing. Interviews were conducted in Dutch by a Masters student trained in qualitative research methods. Interviewees received no reimbursement for their participation. For a topic list see Additional file 2. All identifiable information was removed from the transcripts. No information on age or education level was collected from interviewees.

#### Focus groups

Two sets of two focus groups (one on alcohol, one on smoking) were held in March 2016 and again in June 2016; before and after a concept version of the digital MyCourse interventions was developed. The first two focus groups mainly discussed experiences with quitting smoking or moderating alcohol use and preferences for how to receive support while quitting/moderating use. The last two focus groups mainly discussed themes that should be addressed in the interventions, ways to target cancer survivors without stigmatizing them, preferred tone-of-voice of the texts of the interventions and they could provide feedback on concept versions of the interventions. Focus groups lasted approximately 2 h including a short break. All focus groups were conducted at the Trimbos Institute, which is centrally located in The Netherlands and participants received a €30 gift card for their participation and reimbursement for their travel costs. At the start of each focus group a paper and pencil Timeline Follow Back (TLFB) questionnaire was filled out by the participants to assess participants’ tobacco and alcohol use. TLFB is an assessment tool that obtains estimates of substance use in the past 7 days [16]. Focus groups were conducted by a masters student and AMu. Written informed consent was obtained at the start of the focus group. For a topic list see Additional file 3.

#### Experts

We interviewed 6 experts in April and May 2016 and held a face-to-face expert meeting in May 2016 at the Trimbos Institute. Experts received no reimbursement. During the meeting, results from the survey and findings from the first round of focus groups and interviews were discussed. For a topic list see Additional file 4. All identifiable personal information was removed from the transcripts before the analysis.

### Data analyses

#### Quantitative analysis

Descriptive statistics are provided for the survey sample. Chi-square tests were used to assess any differences between male and female participants and different education levels, applying a conventional statistical significance level (α ≤ 0.05). All survey responses were analysed with R version 3.5.1 [17].

#### Qualitative analysis

Interviews, focus groups and the expert meeting were all recorded and transcribed verbatim. The analysis of the transcripts was carried out according to the Framework method [18]. Authors AMu and DY both individually double coded all transcripts. In addition to these transcripts, responses to one open-ended question from the survey were thematically analysed: “How does a lifestyle intervention for cancer survivors differ from a lifestyle intervention for the general public?” After immersion with the transcripts, an inductive process of open coding was started. Each analysed transcript was discussed in periodic meetings between AMu and DY, and any discrepancies were resolved. Next, these codes were analysed and categorized into broader sub-themes, again using an inductive approach. Afterwards, transcripts were compared to the coding schemes and sub-themes, to ensure robustness of the analysis process and to confirm that all data were reflected in the coding. The main findings were summarised and clustered into larger themes. Authors BB and MB each read and compared two manuscripts to the themes and sub-themes and checked for any missing sub-themes or connections. Software program MaxQDA 18.2.3 was used for management of the transcripts and coding schemes [19]. All quotes provided in this article were translated from Dutch into English. To ensure anonymity any personal information was removed from quotes.

## Results

### Quantitative analysis

#### Sample characteristics

The final survey sample consisted of 240 participants. Majority of the participants were female (*n* = 151, 62.9%), had a high (*n* = 117, 48.8%) or middle level education (*n* = 79, 32.9%), and had been treated for cancer in the past, but were not currently receiving treatment (*n* = 124, 51.7%). Mean age of the sample was 54.9 years (SD = 10.56, range = 20–77 years). Participants were diagnosed with a broad range of types of cancer, most often breast cancer (27.5%) and lymph node cancer or leukaemia (15.8%). See Table 1 for detailed sample characteristics. All but three participants reported having ever used the Internet to find information on health or health care (*n* = 237, 98.8%).

**Table 1:** Demographic characteristics of survey participant sample

Characteristics	Full sample (*n* = 240) n (%^a^)
**Age (years)(mean, SD)**	54.91 (10.56)
**Gender**	
Female	151 (62.9%)
**Education level**	
High level education	117 (48.8%)
Middle level education	79 (32.9%)
Lower level education	38 (15.8%)
No qualifications	1 (0.4%)
**Self-reported cancer status**^**b**^	
I have cancer	83 (34.6%)
I am currently undergoing treatment for cancer	83 (34.6%)
I have been treated for cancer in the past, but not anymore	124 (51.7%)
**Cancer type**^**c**^	
Breast	66 (27.5%)
Lymphatic or leukaemia	38 (15.8%)
Bowel or colon	30 (12.5%)
Bladder or urinary tract	19 (7.9%)
Prostate	16 (6.7%)
Head and neck	16 (6.7%)
Rather not say	1 (0.4%)
Other^d^	68 (28.3%)

*Note*. High level education: bachelor’s or master’s degree. Middle education: four to six years of high school education or secondary vocational education. Lower education: three years of high school or less. ^a^ Due to rounding and some missings, percentages may not add up to 100%. ^b^ Some participants identified with multiple statuses. ^c^ Some participants were diagnosed with multiple types of cancers. ^d^ Other types include: stomach, multiple myeloma, skin, lung, pancreas, oesophagus, endometrial, ovary and more

#### Alcohol and tobacco use in survey sample

Most participants were former smokers (*n* = 132, 55.0%). Current smokers (*n* = 29, 12.1%) mostly smoked 1–10 cigarettes a day (*n* = 13, 44.8%) and the majority of all smokers considered quitting smoking (*n* = 23, 79.3%). A large majority of the participants drank alcohol at the time of the survey (*n* = 186, 77.5%). They most often drank at least 2 times a week (*n* = 99, 53.2%) with at least 3 standard drinks per drinking day (*n* = 176, 94.6%) and most did not consider moderating or quitting their alcohol use (*n* = 158, 84.9%). Most participants never drank more than 6 glasses a day (*n* = 118, 63.4%), followed by those that did so less than once a month (*n* = 49, 20.4%). A minority drank more than 6 glasses a day monthly (*n* = 10, 5.3%), weekly (*n* = 6, 2.5%) or (almost) daily (*n* = 3, 1.3%). See Table 2 for alcohol and tobacco use outcomes. Tobacco use differed between age groups (χ^2^ = 21.39, *P* < .01); participants over 55 years old were more often former smokers, while those aged between 20 and 45 years old were more often never smokers. Drinking status differed between women and men (χ^2^ = 9.38, *P* < .01); women being more often never-drinkers than men, and men more often being current drinkers.

**Table 2:** Alcohol and tobacco use in survey participant sample

Variable	n (%^a^) *N* = 240
**Smoking status**	
Current smoker	29 (12.1)
Former smoker	132 (55.0)
Never smoker	78 (32.5)
**Number of cigarettes per day**	
1–10	13 (44.8^b^)
11–20	8 (27.6^b^)
21 or more	4 (13.8^b^)
0, I smoke incidentally	4 (13.8^b^)
**First cigarette, in minutes after waking up**	
Within 5 min	3 (10.3^b^)
6–30 min	13 (44.8^b^)
31–60 min	7 (24.1^b^)
More than 60 min	6 (20.7^b^)
**Considering to quit smoking**	
Yes	23 (79.3^b^)
No	6 (20.7^b^)
**Drinking status**	
Current drinker	186 (77.5)
Former drinker	29 (12.1)
Never drinker	24 (10.0)
**Frequency of drinking**	
Never	1 (0.5^c^)
Once a month or less	31 (16.7^c^)
2 to 4 times a month	55 (29.6^c^)
2 to 3 times a week	51 (27.4^c^)
4 or more times a week	48 (25.8^c^)
**Standard units on a drinking day**^**d**^	
1 or 2	141 (75.8^c^)
3 or 4	35 (18.8^c^)
5 or 6	7 (3.8^c^)
7 or more	3 (1.6^c^)
**Considering to quit or moderate drinking**	
Yes	27 (14.5^c^)
No	158 (84.9^c^)

*Note*. ^a^ Due to rounding and missings, percentages may not add up to 100. ^b^ Proportion of current smokers, total *n* = 29. ^c^ Proportion of current drinkers, total *n* = 186. ^d^ 1 standard unit = 10 g ethanol

### AM and SC in survey sample

Alcohol use was discussed during treatment with only 21.3% (*n* = 51) of all participants and 19.4% (*n* = 36) of current drinkers, tobacco use was discussed with 40.4% (*n* = 97) of the total sample and 69.0% of current smokers (*n* = 20). Discussion of alcohol use was not associated with drinking status (χ^2^ = 5.11, *P* = .08). Discussion of tobacco use was associated with smoking status (χ^2^ = 22.5, *P* < .01).

Of those participants drinking at the time of diagnosis (*n* = 200, 83.3%), almost half had not tried to moderate their alcohol use before, during or after cancer treatment (*n* = 85, 42.5%), those who did, mostly did so during treatment (*n* = 70, 35%). A fourth of participants reported smoking at the time of their cancer diagnosis (*n* = 59, 24.6%). Participants most often attempted to quit or moderate smoking before treatment started (*n* = 26, 44.1%), followed by during treatment (*n* = 17, 28.8%), and a minority attempted to quit after the treatment phase (*n* = 11, 18.6%).

Reasons for quitting or moderating alcohol use were mostly related to health (*n* = 57, 44.2%) and cancer treatment (e.g., not being able to tolerate alcohol during treatment) (*n* = 6, 4.7%). No one reported advice from a doctor as a reason for AM. Most often reported reasons for not quitting alcohol use were: one’s alcohol use reportedly being limited and not deemed problematic (*n* = 72, 38.7%), alcohol being enjoyed and enjoyment valued as important (*n* = 31, 16.7%), and not seeing the benefits of quitting at this point (*n* = 27, 14.5%). Remarkably, 6 participants reported that alcohol use benefited their health or that a doctor had encouraged them to keep drinking (moderately). Most frequently reported reason for trying to quit smoking was also related to health benefits (*n* = 36, 66.7%). Current smokers most often reported having tried, but not being able to quit as a reason for not quitting (*n* = 9, 31.0%). Two participants reported that they were told by a doctor that SC was unnecessary if they kept it at a few cigarettes a week or that SC would cause so much stress that it would be more detrimental to treatment.

#### Preferences for support

Current and former smokers and drinkers preferred different types of support (see Additional file 5. Nearly half of (former) drinkers and about a third of (former) smokers did not want any support (*n* = 100, 46.5% and *n* = 58, 36.0%, respectively). Most preferred options among those who wanted support were: online information, a printed information flyer, and face-to-face support. A free online self-management course was preferred most by current smokers (*n* = 5, 17.2%) and least by former drinkers (*n* = 1, 3.4%). Face-to-face support was preferred more for SC (14.3%) than for AM (*n* = 14, 6.5%).

When asked about whom they would like to be supported by, many did not want support from anyone for AM (*n* = 101, 47.0%) or SC (*n* = 67, 41.6%). For both AM and SC, support from family (AM: *n* = 25, 11.6%; SC: *n* = 35, 21.7%) and a coach (AM: *n* = 29, 13.5%; SC: *n* = 33, 20.5%) was most preferred.

### Qualitative analysis

#### Sample characteristics

Eight cancer survivors and six experts were interviewed. Cancer survivors were a convenience sample recruited using social media and snowball sampling. They were either smokers, former smokers or currently using alcohol. Interviewed experts were researchers with experience in development and implementation of digital support (eg for emotional well-being) for cancer survivors (*n* = 4) and healthcare professionals experienced in supporting SC and AM (*n* = 2).

Focus groups consisted of 6, 5, 3 and 6 participants respectively. Five participants attended two focus groups, before and after the development of a concept intervention, resulting in a sample of 15 unique focus group participants. The expert meeting (*n* = 7) was attended by researchers from the Trimbos Institute with experience in developing digital lifestyle or depression prevention interventions.

All cancer survivor interviewees (*n* = 8) and focus group participants (*n* = 15) were adults diagnosed with cancer at some point in their lives. Majority of the interviewees and focus group participants were highly educated. Interviewees were diagnosed with a broad range of types of cancer, see Table 3 for more characteristics. Below, we present the main themes that were extracted from the interviews and focus groups.

**Table 3:** Demographic characteristics of cancer survivors in focus groups and interviews

Characteristics	Focus groups (*N* = 15) n	Interviews (*N* = 8) n
**Gender**		
Female	5	5
**Education level**		
High level education	11	–
Middle education	3	–
Lower education	1	–
**Smoking status**		
current smoker	3	6
non-smoker^a^	3	0
former smoker	9	2
missing	0	0
**Drinking status**		
current drinker	13	4
currently non drinker	2	2
missing	0	2
**Type of cancer**		
breast	3	1
melanoma	1	3
prostate	3	0
non-Hodgkin	3	0
other^b^	5	4

*Note.* High level education: bachelor’s or master’s degree. Middle education: four to six years of high school education or secondary vocational education. Lower education: three years of high school or less. ^a^ Unknown whether they are a former or never smoker. Other reported cancers were: cancer of the bladder, colon, oesophageal, stomach, lung, Hodgkin’s lymphoma, and head and neck cancer

### Cancer survivors’ perspectives on AM and SC

#### Identification as cancer survivors

The time from cancer diagnosis to treatment and afterwards was described by almost everyone as impactful, although for each different aspects stood out; ranging from the shock of the diagnosis, severity of the treatment phase and physical limitations, to worries about job security and the multitude of instructions on what (not) to do, for example what to eat. Often repeated is the newly developed lack of trust in the body, causing them to be vigilant at aches or pains.

“Having cancer has a great impact and affects many aspects of your life. Recognizing and understanding this is key in a lifestyle intervention.” [*survey participant P81, colon cancer survivor, female, current drinker*]

“You see, before that I could trust my body, sometimes I felt something, but I was convinced it would pass. But now whenever I feel something, I go: ‘Oh no, you don’t think it’s … ’ It’s a different type of life, really.” [*focus group participant FS2A, non-Hodgkin cancer survivor, male, current smoker*]

Notwithstanding this impact, many would not identify as ‘cancer survivors’, rather feeling better addressed by the terms: ‘people who have had cancer’ or ‘people with cancer’. They would most like to move on from that part of their lives, only identifying as a cancer survivor when a doctor’s appointment comes up:

“There are many periods that cancer is not on my mind at all. But then an appointment date for a control or scan comes up and slowly I start to become aware of it.” [*focus group participant FS1B, prostate cancer survivor, male, former smoker*]

Furthermore, when asked about the differences between a lifestyle program for the general population and for cancer survivors, many responded surprised and stated there ‘should be no difference’. Survey responses showed that this partly reflects a fear of stigmatization and that being labelled as cancer survivors is seen as something undesirable.

“You receive a stamp, the ‘cancer-stamp’, well you definitely don’t want that.” [*interviewee C9, melanoma cancer survivor, female, former smoker, current drinker*]

The reluctance to identify as cancer survivors combined with the view that SC and AM are important to all people, not just cancer survivors, translates into not actively seeking out programs specific to cancer survivors, but turning first towards general (health) programs. Websites specific to cancer survivors were feared to be too confronting and that they could also be at odds with the desire for a positive tone of voice, because of negative associations with cancer. However, cancer survivors were triggered when reading something about their specific type of cancer (e.g., breast cancer or colon cancer). Possible benefits of an intervention aimed at cancer survivors were also mentioned: information on how AM and SC influence cancer, a safe space to discuss issues with peers (e.g., smoking after lung cancer) or extra emotional support. The need for a cancer specific website seemed to depend on the time since diagnosis, a more recent diagnosis indicating a greater need:

“If I would use a program, I would use the one for the general population, because for me much time has passed since [the diagnosis]. [ … ] the moment you get out of treatment or when you’re in the middle of it, there is still so much going on. You’re psychologically less stable, you experience many emotions, you have more physical complaints, apart from the withdrawal symptoms. [ … ] So I do think in that case it should be combined.” [*focus group participant FA2B, Hodgkin cancer survivor, female, former smoker*]

#### Differential beliefs about health consequences

Beliefs about health consequences differ for alcohol and smoking. Participants are generally convinced of their knowledge of the harmful effects of smoking, including the increased cancer risk. Not only because of the available and known evidence, but because they have experienced the detrimental effects of smoking themselves in their coughing or deterioration of their physical condition. Some (former) smoking cancer survivors were convinced of the detrimental effects of smoking, but emphasized their disbelief that their specific type of cancer was caused by smoking.

Participants were far less aware of the harmful effects of alcohol use and its association with cancer. For example, each focus group on AM started with questions from the participants on the harmful effects of alcohol use, while no such questions were asked about smoking. Specifically, it was unclear what amount of alcohol is detrimental, what influence alcohol has on cancer treatment and recurrence, and there was confusion about the benefits of alcohol on the cardio-vascular system, as communicated in previous years.

#### Differential beliefs about enjoyment and relaxation

The importance of enjoying life and of relaxing was emphasized throughout all responses, interviews and focus groups, shaping views on alcohol use and smoking. Smoking was seen as a means to calm down, destress and relax. For some cancer survivors it was the only way they knew how to cope with negative emotions or feelings of stress and anxiety. Although alcohol was used to handle negative feelings as well, more often it was associated with enjoyment, having a good time and relaxation. It was seen as an important part of a lifestyle. A smoking cancer survivor explained the value smoking had for them:

“[when asked about smoking] This is my buddy and it’s always there for me. To comfort me, or to keep me company, but it’s always there for me.” [*interviewee C3, colon cancer survivor, female, current smoker*]

#### Willingness to quit

These differential beliefs about smoking and alcohol use are reflected in the willingness to quit or moderate. Most former and current smokers in focus groups and in the survey, believed that all smokers would like to quit, but might be unable to or afraid. For example, one participant reported thinking about SC regularly, and she wanted to understand why, after her cancer diagnosis, she kept on smoking. However, interviewed smoking cancer survivors mostly reported not wanting to quit at all or showed ambivalence towards quitting, reporting that their wish was based on wanting to please others. They generally acknowledged and recognized their addiction. One interviewee stated after many quit attempts in the past:

“It doesn’t bother me anymore and I don’t feel the need to quit anymore. There are not that many fun things in my life anymore. [ … ] It’s a very useless motion, it’s nothing, but to me it means a lot.” [*interviewee C3, colon cancer survivor, female, current smoker*]

Concerning alcohol use, only one participant reported trying to quit alcohol use and not succeeding, whereas for SC many failed attempts were reported. Generally, cancer survivors stated they would be able to quit their alcohol use easily if they were convinced of its detrimental effects. A doctor’s advice was valued highly in this regard; a doctor’s recommendation to quit drinking would make them consider moderation. Importantly, alcohol use was rarely addressed by healthcare professionals, in contrast to smoking, when cancer survivors would have expected and valued it. As only few participants were convinced of alcohol’s detrimental effects, or saw it as less harmful than other detrimental behaviours, alcohol use was mostly viewed as an important aspect of their lifestyle that they did not consider changing:

“I’m still in the middle of this whole cancer story, so how amazing is it that in the evening I get to sit back, grab a piece of cheese and a glass of port [alcoholic beverage]. Why would I want to moderate that. On the other hand, I do think that you should do anything you can to keep your body and condition in shape. [ … ] So I drink my glass of beer, but I also go to the gym. That’s how I keep life a little fun. [ … ].” [*focus group participant FA2A, prostate cancer survivor, former smoker*]

#### Autonomy is key

A prominent theme was the need for autonomy; making one’s own informed decisions and being as independent as possible. This need became evident in different ways. Healthcare professionals’ lifestyle advice was valued highly, but should leave room for the patient’s own decision on how to apply it to their personal circumstances: being able to set their own goals and pace, e.g. smoking less, even if they were not completely in line with current guidelines. Regarding peer support, cancer survivors would like to stay as independent as possible, not relying too heavily on others and not burdening them. The central role of autonomy became most clear when discussing motivations for behaviour change.

In many different wordings, the importance of something we have named ‘intrinsic motivation’ for AM and SC was stressed: “taking responsibility”, “willpower”, “coming from the inside out”, “my own wish”, “supporting your own decision”. Participants made a distinction between undertaking AM and SC efforts to please others (e.g., partner, family or doctor), or out of their own will to quit smoking or moderate their drinking. The first was related to failed quit attempts, whereas the second was seen as a prerequisite for AM and SC.

“It needs to come from within yourself, if you don’t have the willpower, then it’s never going to work.” [*focus group participant FA2C, eye melanoma cancer survivor, male, former smoker*]

“I would grant it myself, but that moment might come and then it would need to come from within me. Or it might not.” [*interviewee C4, lung cancer survivor, current smoker*]

It remained unclear how to achieve intrinsic motivation, but cancer survivors provided several insights. Experiencing short term benefits and seeing results of AM or SC increased intrinsic motivation. A crucial insight was that being convinced of the health benefits of AM and SC, although important, was not enough for intrinsic motivation, because bigger concerns might be at play (e.g., ways to destress) or other things might be valued more (e.g., enjoyment). Lastly, some kind of ‘momentum’ should be used; capitalizing on moments when someone is receptive of lifestyle behaviour change. Cancer survivors differed in opinion on whether intrinsic motivation is needed before engaging in digital programs and other types of support, or whether these support programs might help to increase intrinsic motivation in an ambivalent phase.

Extrinsic motivation is not without importance, as illustrated by the fact that for many participants advice by a healthcare professional influenced SC or AM efforts. Furthermore, seeing others’ achievements could be motivating for some. But extrinsic motivation needed to be translated into an intrinsic will in order to have a real and lasting impact on behaviour change.

Too great of an emphasis on intrinsic motivation could hinder quit attempts. Relating in particular to SC, some participants strongly stated that “with the right mentality anyone can quit cold turkey”, thus confirming the detrimental, but persistent belief that SC is only a matter of firmly deciding not to smoke which leads many to not seek SC support in their quit attempts.

### Cancer survivors’ preferences regarding digital AM and SC support

#### Tone of voice

An important theme that arose is the clear and explicit need for a positive, non-condemning and non-patronizing tone of voice. Positivity in the sense that there should be emphasis on complimenting accomplishments, rather than on criticizing what went wrong, emphasis on what is to be gained from AM or SC, rather than listing diseases with increased risk, and emphasis on what cancer survivors can do (better), rather than what they are limited in.

“For me, it’s the approach. ‘Oh you managed to only have one [cigarette].’ Very much focused on your success, on your strength and not on judgement. Judgement is catastrophic for me, I will only smoke more.” [*interviewee C4, lung cancer survivor, female, current smoker*]

Some cancer survivors who use tobacco or alcohol had to deal with judgemental reactions from their social networks, as if they “had brought it on themselves”. This could result in feelings of guilt: *“I start a small fight with myself and think: ‘well there you go again, you’re smoking again.’ You’re judging yourself, but then I also think: ‘well it’s really very nice.’*” *(interviewee, breast cancer survivor, female, current smoker and drinker).* Therefore a non-condemning tone-of-voice is important. A difference was perceived in societal reactions to alcohol use and smoking. Alcohol use after cancer was seen as more acceptable, whereas smoking cancer survivors often felt directly judged when smoking. On the other hand, dependency on alcohol was seen as more severe than an addiction to cigarettes, which was considered more ‘normal’.

Concerning AM, participants did not want to be labelled as alcoholics or alcohol misusers, as they did not identify with those labels, but simply as people who wanted to moderate their alcohol use. Content should emphasize that cancer survivors can make their own decisions. A conversational tone of voice (e.g., asking about the nature of one’s alcohol use) was preferred over only presenting alcohol’s adverse effects, which was considered patronizing. The tone-of-voice was perceived as an important barrier for receiving information about or engaging in AM or SC interventions, as illustrated by a participant looking for information online:

“There was this website, saying that it’s my fault that I got cancer. [ … ] I read about oesophageal cancer and the combination of alcohol and smoking, and I don’t know what else they dragged into it. Then I looked up some other cancer types that are not related to it, and well, then that means the whole world is living wrongly. I messaged them: ‘I am a cancer patient and you are saying that it’s my fault that I have cancer, and I object to that.’” [*focus group participant FA1A, oesophageal cancer survivor, male, non-smoker*]

#### Specific intervention components

Preferences for specific intervention components were discussed, of which we highlight the most notable ones in Table 4, showing that cancer survivors are a diverse group resulting in differing preferences.

**Table 4:** Preferences concerning specific intervention components and considerations for implementation

Component	Preferences	Implementation considerations
Monitoring of alcohol or tobacco use	It is experienced as offering insight into drinking and smoking patterns. However, for some it could be too confronting, especially when goals of moderation or cessation are not met, leading people to not report drinking or smoking truthfully.	This emphasizes the need for an accepting, non-judgemental tone-of-voice throughout the program.
Peer support	Some take great support from it and emphasize benefits such as a better understanding of the cancer experience and the possibility to talk in a light-hearted way about the cancer experience. Whereas others had experienced that forums often contain negative experiences or unverified information, invoking negative emotions and worries.	Cancer survivors suggest to incorporate peer support in a non-prominent way, offering cancer survivors the choice to either engage with it or not and include monitoring of the platform to prevent the spread of false information.
Involvement of own social network (family and friends)	A clear preference for a supportive role instead of a correcting role: preference for compliments for SC or AM efforts and implicit support such as not offering cigarettes or alcohol, but not repeatedly asking whether someone had smoked or how many drinks they had had.	The social network does not always know how to best support cancer survivors or SC and AM efforts. At the same time, cancer survivors can be hesitant to let people help, recognizing the impact of the cancer experience on their family and friends.
Moment of addressing AM or SC	Some would like AM and SC addressed at the start of treatment because then they see its potential benefits, but others would only be receptive to it after finishing the treatment phase, as they have too many things on their mind during treatment.	Flexibility in moment of addressing SC or AM.
Digital delivery mode	Essential to a digital program would be the protection of personal data, not fearing that anyone but the patients themselves could get hold of their data. It should be easy to use, on both smartphones and tablets, and it should be inviting during the most difficult moments of AM and SC.	Guidance, regular updates and interactive content could help motivate use of the intervention.

Focus group participants were all comfortable using the Internet, but some interviewees reported rarely using the Internet, or not using it for information on cancer and health because it distresses them. This last group preferred face-to-face contact over completely digital support. Cancer survivors who were comfortable using the Internet were concerned about the self-discipline and willpower that they believed digital programs require. Guidance from a healthcare professional, researcher or peers could motivate continued program use. Those who were interested in a digital program, reasoned that they “would grant themselves a try” or see it as “a stepping stone”. Other perceived benefits were the flexibility to use a digital program at any place and time, and that digital programs do not require extra hospital visits.

### Experts’ recommendations for successful AM and SC interventions for cancer survivors

A major concern from experts was drop-out and shorter than intended use of a digital intervention. Experts recommended to clearly communicate what benefit is to be realistically expected from a digital intervention, possibly in person at the start of the intervention. Patients who have severe alcohol or tobacco dependence should be alerted to the fact that a digital intervention might offer insufficient support and they should be referred to additional support.

Experts also emphasize the importance of autonomy and intrinsic motivation. A personal and tailored approach could help achieve this motivation. To engage with digital interventions, greater levels of motivation and self-discipline were believed to be necessary:

“We see a lot of working people, I feel like they appreciate getting to choose when they want to engage with treatment. On the other hand, the downside is that people really need self-discipline to use the program regularly.” [*interviewee E2, eHealth developer and healthcare professional*]

Interventions aimed at cancer survivors should take into account pronounced fatigue and mood problems, emphasize the benefits of AM and SC for treatment and recovery, and address different coping mechanisms to deal with increased anxiety. For AM specifically, problem recognition is an issue. Most people do not identify with the general image of problematic alcohol use, even if their alcohol use is well above recommended guidelines. Healthcare professionals are in a unique position to relate health outcomes or symptoms to smoking or alcohol use, thereby increasing awareness of their health consequences and create intrinsic motivation to change drinking behaviour:

“And then I say: ‘Your palpitations and stress might be related to the way you are handling it [by drinking].’ And that’s when he said: ‘Yes, I should handle it differently and play more tennis with someone.” [*interviewee E4, healthcare professional*]

Concerning AM in particular, it should be taken into account that realizing the extent of their alcohol problems can be uncomfortable for a patient due to the stigma surrounding alcohol misuse and alcohol dependency.

Experts also mentioned referral to the digital interventions by healthcare professionals as an important implementation route, because their advice is valued by cancer survivors. For healthcare professionals, it is a benefit to be able to offer tangible digital interventions as an extra tool while addressing AM or SC. However, one healthcare professional reported that the vast amount of available SC and AM apps and uncertainty about which would work (best) for the patient was a barrier for referral.

## Discussion

We aimed to explore cancer survivors’ views on AM and SC, their preferences regarding digital support for AM and SC, and experts’ recommendations for successful AM and SC interventions for cancer survivors. This information was used to inform the development of SC and AM digital interventions [15], considering that digital interventions may provide a low threshold for seeking help and reach a large population and that they have shown their effectiveness in the general population [6, 7]. Our findings are also largely in line with findings from previous qualitative studies on health behaviour change and SC. Only limited findings were available on AM in cancer survivors. In the section below several behavioural models and theories provide a framework to understand and integrate our key findings.

### What are cancer survivors’ views on SC and AM?

Views on AM differed from views on SC in several ways: awareness of the health consequences was higher for smoking than for alcohol drinking, smokers more often considered quitting smoking, and for AM more low-intensity support was preferred. Enjoyment and relaxation were associated with both smoking and drinking and provided a barrier for AM and SC. Intrinsic motivation was deemed essential for successful quit attempts.

Majority of current smoking cancer survivors in the survey considered quitting, while most current drinkers did not consider moderation. This lack of interest in AM might be driven by low recognition of problematic alcohol use, alcohol use being more socially acceptable and the lack of awareness of the health benefits of AM or the association between alcohol and cancer. The way these different normative beliefs can influence the attitude towards AM or SC is predicted by the Theory of Planned Behaviour, which describes six constructs that influence intentions for behaviour change: behavioural beliefs, attitude toward the behaviour, normative beliefs, subjective norm, control beliefs and perceived behavioural control [20]. Furthermore, SC was perceived as difficult (perceived behavioural control) and seemed to lead to decreased SC efforts, despite statements of their awareness of health benefits of SC. For AM, most participants believed they could easily moderate alcohol use if they wanted to. The preference for low-intensity AM support as found in this study, could reflect both confidence in being able to moderate (perceived behavioural control) and the low problem recognition of problematic alcohol use (attitude). Cognitive Behavioural Therapy could help participants understand the associations between cognitions (beliefs, attitudes) and smoking or drinking behaviour, and it could influence perceived behavioural control by teaching skills to cope with feelings and situations that make SC or AM difficult [21].

Heightened awareness of their health can make cancer survivors more receptive to health messages of AM and SC [11, 22]. But in some, the increased valuation of enjoying life following their cancer diagnosis can make them reluctant to moderate alcohol use or quit smoking. Smoking was also previously found to be a source of enjoyment and way of psychological coping that is highly valued [8, 10]. Still others did not see the benefit of quitting or moderating after cancer. This lack of motivation for SC or positive health behaviour change was noted earlier as well [8, 10]. Increased feelings of stress or anxiety can also contribute to the reluctance to quit smoking, because smoking has been a way of coping with negative feelings. Motivational Interviewing could help participants identify and solve ambivalence towards SC or AM [23].

### What are cancer survivors’ preferences regarding digital support for SC and AM?

There was great variety in cancer survivors and their experiences, and this was reflected in different practical preferences for digital AM and SC support (see Table 4) as well as different views on interventions specifically for cancer survivors. However, all participants shared the need for a positive, non-judgemental tone-of-voice that emphasized autonomy, possibilities for personalization and emphasis on short-term benefits. Face-to-face support should be available to those not interested in digital programs, for example because they are not comfortable using the Internet for health-related topics.

Not all cancer survivors would feel addressed by an intervention aimed solely at cancer survivors and might prefer an intervention for the general population, for fear of stigmatization or because of the desire to move on from the cancer diagnosis. Tailoring to this population therefore has to be done subtly, for instance by not presenting AM or SC programs as a program only for cancer survivors, but using targeted implementation strategies to reach cancer survivors. MyCourse was developed for cancer survivors specifically, to make it easier for the target population to find the intervention and as the need for a cancer-specific website may be greater around the time of diagnosis. Cancer survivors preferred information on short-term benefits of AM and SC (in contrast to long term prevention of disease), as these directly influence their daily life and are thus perceived more relevant. This is predicted by the elaboration likelihood model which postulates that presentation of highly relevant information will encourage behaviour change (Petty & Cacioppo, 1986).

Our study showed that pivotal throughout all communication about AM and SC is recognizing and emphasizing the autonomy of the cancer survivor; one way to do so is by adopting a non-patronizing, non-condemning, positive tone-of-voice. Healthy lifestyle recommendations should be presented as advice and there should be explicit room for people to choose in what way to adhere to these guidelines (e.g., leaving it to the client to decide on drinking pattern goals, and not only offer the choice to either quit or reduce to national drinking guidelines). The importance of autonomy is in line with previous findings, where it is referred to as ‘personal control’ [9].

The importance of intrinsic motivation for SC and AM was repeatedly noted, and failed quit attempts for SC were related to wanting to please others instead of being intrinsically motivated. Breast cancer survivors meeting physical activity guidelines actually reported higher intrinsic motivation and greater perceived autonomy support [24]. Self-determination Theory (SDT) helps understand the recurring emphasis on autonomy; it postulates that autonomy is one of the basic psychological needs, next to competence and relatedness, whose satisfaction encourages development of intrinsic motivation [25]. The more intrinsic motivation for behaviour change is, the higher the likelihood to change and persist in the behaviour, according to the theory.

Cancer survivors in the current study did not know of ways to encourage intrinsic motivation, but several of their recommendations and preferences in reference to other topics were in line with what SDT refers to as an autonomously supportive context (or encouraging a sense of autonomy, relatedness and competence), which fosters development of human’s natural tendency to intrinsic motivation: positive performance feedback, absence of controlling remarks from their social network, freedom to determine one’s own goals (for AM or SC) and acknowledgement of their feelings (either in peer support groups or their social network). Acceptance and commitment therapy (ACT) could help encourage intrinsic motivation, as it focuses on participant’s values that guide them to the desired behaviour of AM or SC [26].

### What are experts’ recommendations for successful digital AM and SC support for cancer survivors?

Experts emphasized raising awareness on alcohol-related harm and alcohol problem recognition, taking into account pronounced fatigue and mood problems, and the need for engagement of healthcare professionals. They expressed concern about drop-out. The lack of discussion of SC with healthcare professionals and simultaneously the willingness to address it corroborates previous findings [8, 9]. It should be noted that this study showed that addressing SC and AM by healthcare professionals is lacking, especially for AM, but that it would be valued by cancer survivors.

### Strengths and limitations

This study used a variety of inductive methods to assess perspectives on support for SC and AM in cancer survivors: a survey, focus groups and interviews among cancer survivors, a meeting and interviews with experts in the field of eHealth, AM, SC and cancer survivors. These methods all complemented each other. By including eHealth experts and not only healthcare professionals, a perspective on the specific requirements for digital AM and SC interventions was provided. There was great variation in cancer types and treatment phases in participants. Limitations include that the recruitment of most participants took place through an online cancer patient platform. This selection might have influenced results on preferences for support, as participants probably enjoy spending time online and actively seek online information. Interviewed participants were, however, not all members of the online platform and at times provided a different point of view (e.g., more often reported lack of willingness for AM or SC than focus group participants). The difficulties in recruiting participants for the qualitative part of the study might point to the intricacies of addressing AM and SC in cancer survivors and possibly the lack of awareness around the importance of these topics. The survey results cannot be generalized to all Dutch cancer survivors, as the survey was not a representative sample (e.g., it included mostly higher educated participants and women). It is possible that in a survey sample consisting of more excessive drinkers, more respondents would consider alcohol moderation, than was found in the current sample.

### Clinical implications

The differences between perspectives on AM and perspectives on SC found in this study have implications for public health messages and approaches to AM and SC by healthcare professionals. Care should be taken to explain the relationship between alcohol, health and cancer. For SC, it could be promising to address the fear of quitting and replacement of the cigarette as a means for relaxation and keeping busy. Emphasizing that there does not have to be a relation between smoking or alcohol and the cause of someone’s cancer, and that AM and SC have other (short-term) benefits, could help avoid feelings of guilt. It is important to encourage healthcare professionals to address AM and SC with cancer survivors, and digital self-help interventions can be a useful tool to refer patients to.

### Future research

Future research should study how healthcare professionals could be encouraged to routinely address AM and SC in cancer survivors. Because of the fear of stigma and great variety in cancer types, different ways should be explored to efficiently tailor interventions to appeal to cancer survivors without stigmatizing them. More research should be conducted into ways to (intrinsically) motivate cancer survivors who are currently unwilling or ambivalent towards AM or SC.

## Conclusion

Our study findings shed light on how cancer patients view AM and SC support, and informs the development of digital interventions for AM and SC in cancer survivors. To encourage AM specifically, problem recognition and the awareness of the benefits of AM should be addressed, partly by involving healthcare professionals. Although some cancer survivors prefer no support or face-to-face contact, digital AM and SC interventions for cancer survivors are seen as a valuable solution. These interventions should incorporate a positive, non-judgemental and non-patronizing tone-of-voice, address concerns especially relevant to cancer survivors, offer possibilities for personalization and emphasize autonomy throughout. Care should be taken as to avoid stigmatization when tailoring interventions to cancer survivors.

## Supplementary Information

**Additional file 1.** Survey questions translated from Dutch.

**Additional file 2.** Interview topic guide for cancer survivors.

**Additional file 3.** Focus groups topic guide.

**Additional file 4.** Interview topic guide for healthcare professionals and experts.

**Additional file 5.** Table S5. Preferences for support in current users vs former users of alcohol and tobacco.

## Data Availability

The qualitative and quantitative datasets collected during the current study are not publicly available due to their containing information that could compromise the privacy of research participants, but requests to analyse data can be made to the corresponding author.

## Abbreviations

AM: Alcohol Moderation
COREQ: Consolidated criteria for reporting qualitative research
SC: Smoking Cessation
SDT: Self-determination Theory
TLFB: Timeline Follow-Back method
