# Supplementary material for: Cancer survivors’ views on digital support for smoking cessation and alcohol moderation: a survey and qualitative study
Source: BMC Public Health. 2021 Sep 27;21:1763. doi: 10.1186/s12889-021-11785-7 (PMC8477484; doi:10.1186/s12889-021-11785-7)
Supplement: Supplementary file 1 — Additional file 1. Survey questions translated from Dutch. [file 12889_2021_11785_MOESM1_ESM.docx]

**Appendix 1**

**Survey questions translated from Dutch**

What is your gender?

- Male
- Female

What is your age?

<numeric>

What is your highest achieved eductional level?

- No qualifications (No education/lower school)
- Lower level education (three to four years of high school)
- Middle level education (Dutch school level MBO level 2-4, HAVO, VWO)
- High level education (university or college)

In what way do you regularly use the Internet?

- I never use the Internet
- Personal computer (PC) or laptop
- Mobile phone/smartphone
- Tablet
- Other

Have you ever used the Internet to look up information on illness, health or healthcare?

- Yes
- No

What type(s) of cancer have you been diagnosed with?

- Breast cancer
- Prostate cancer
- Bowel/colon cancer
- Skin cancer
- Lung cancer
- Leukeamia or lymphatic cancer
- Bladder or urinary tract cancer
- Endometrial cancer
- Heand and neck cancer
- Ovary cancer
- Stomach cancer
- Pancreas cancer
- Kidney cancer
- I woud rather not say
- Other <free text response>

Do you smoke (sometimes) or do you not smoke at all?

- I smoke sometimes
- I do not smoke anymore but I used to smoke
- I have never smoked
- I do not know

How many cigarettes a day do you smoke?

- 1-10 cigarettes
- 11-20 cigarettes
- 21-30 cigarettes
- 31 or more cigarettes
- 0 cigarettes (I smoke incidentally / sometimes)

How quickly after waking up do you smoke the first cigarette?

- Within 5 minutes
- 6-30 minutes
- 31-60 minutes
- More than 60 minutes

Do you consider quitting smoking?

- Yes
- No

Do you (sometimes) drink alcohol or do you not drink alcohol at all?

- I sometimes drink alcohol
- I do not drink alcohol anymore but I used to drink alcohol
- I have never drunk alcohol
- I do not know

How often do you drink alcohol?

- Never
- Once a month or less
- 2 to 4 times a month
- 2 to 3 times a week
- 4 times or more per week

On a day on which you drink alcohol, how many glasses do you usually drink?

- 1 or 2
- 3 or 4
- 5 or 6
- 7 to 9
- 10 or more

How often are there occasions on which you drink more than 6 glasses of alcohol?

- Never
- Less than once a month
- Monthly
- Weekly
- Daily or almost daily

Do you consider quitting or moderating your alcohol use?

- Yes
- No

Was the importance of drinking little to no alcohol discussed with you during treatment?

- Yes
- No

Have you tried moderating your alcohol use before, during or after your treatment?

- Yes, before the treatment
- Yes, during the treatment
- Yes, after the treatment
- No, I have not tried to change my alcohol use
- No, I did not drink alcohol when I was diagnosed

What is a reason that you did not quit alcohol use?

- I have tried, but I cannot do it/I think I cannot do it
- I have bigger problems than my alcohol use
- I do not think it has any benefits to quit now
- I do not really know how to quit alcohol use
- I become anxious or stressed when I do not drink
- I lose weight when I do not drink
- Everyone in my social environment drinks alcohol
- I can focus better when I am drinking alcohol
- I have moderated my alcohol use and so I do not have any wish to quit
- Other <free text response>

When do you plan to moderate or quit alcohol use?

- Within a month
- Within half a year
- I do not know
- I am not planning to

What was the most important reason for attempting to moderate or quit alcohol use?

- My health
- Because my doctor recommended it
- Because my partner/family wanted it
- For my children’s health
- To save money
- Other <free text response>

What form of information or low-threshold support for alcohol moderation would you appreciate/have appreciated before, during or after treatment?

- I would not have appreciated any information or support
- Information online
- Printed information brochure
- Free online self-management course
- Self-help book
- Contact with peers
- Contact with a healthcare professional through skype or email
- Face-to-face contact with a healthcare professional (for example, at the hospital)
- Other <free text response>

What form of alcohol moderation support of others would you have appreciated?

- I would not appreciate support from others
- Support from my family
- Support from my fiends
- Support from social media contacts
- Support from peers who are also attempting to quit or moderate
- Support from peers who also have (had) cancer
- Support from a healthcare professional or coach
- Other <free text response>

What form of information or low-threshold support for alcohol moderation have you actually used before, during or after treatment?

- I have not used any information or support
- Information online
- Printed information brochure
- Free online self-management course
- Self-help book
- Contact with peers
- Contact with a healthcare professional through skype or email
- Face-to-face contact with a healthcare professional (for example, at the hospital)
- Other

Have you tried to quit smoking before, during or after your treatment?

- Yes, before the treatment
- Yes, during the treatment
- Yes, after the treatment
- No, I have not tried to quit smoking
- No, I did not smoke when I was diagnosed

What is a reason that you did not quit smoking?

- I have tried, but I cannot do it/I think I cannot do it
- I have bigger problems than smoking
- I do not think it has any benefits to quit now
- I do not really know how to quit smoking
- I become anxious or stressed when I do not smoke
- I gain weight when I do not smoke
- Everyone in my social environment smokes
- I can focus better when I smoke
- I have greatly reduced my smoking and so I do not have any wish to quit
- Other <free text response>

When do you plan to quit smoking?

- Within a month
- Within half a year
- I do not know
- I am not planning to

What was the most important reason for attempting to quit smoking?

- My health
- Because my doctor recommended it
- Because my partner/family wanted it
- For my children’s health
- To save money
- Other <free text response>

What form of information or low-threshold support for smoking cessation would you appreciate/have appreciated before, during or after treatment?

- I would not have appreciated any information or support
- Information online
- Printed information brochure
- Free online self-management course
- Self-help book
- Contact with peers
- Contact with a healthcare professional through skype or email
- Face-to-face contact with a healthcare professional (for example, at the hospital)
- Other <free text response>

What form of smoking support of others would you have appreciated?

- I would not appreciate support from others
- Support from my family
- Support from my fiends
- Support from social media contacts
- Support from peers who are also attempting to quit or moderate
- Support from peers who also have (had) cancer
- Support from a healthcare professional or coach
- Other <free text response>

What form of information or low-threshold support for smoking cessation have you actually used before, during or after treatment?

- I have not used any information or support
- Information online
- Printed information brochure
- Free online self-management course
- Self-help book
- Contact with peers
- Contact with a healthcare professional through skype or email
- Face-to-face contact with a healthcare professional (for example, at the hospital)
- Other <free text response>

How does a lifestyle intervention for cancer survivors differ from a lifestyle intervention for the general public?

<free text response>
