# Supplementary material for: Cancer survivors’ views on digital support for smoking cessation and alcohol moderation: a survey and qualitative study
Source: BMC Public Health. 2021 Sep 27;21:1763. doi: 10.1186/s12889-021-11785-7 (PMC8477484; doi:10.1186/s12889-021-11785-7)
Supplement: Supplementary file 2 — Additional file 2. Interview topic guide for cancer survivors. [file 12889_2021_11785_MOESM2_ESM.docx]

**Appendix 2**

Interview topic guide for cancer survivors

- Context – participant’s experience of cancer and its influence on their daily lives
- Smoking behaviour – smoking history, feelings and beliefs about smoking, willingness to quit, quit attempts
- Drinking behaviour – drinking history, feelings and beliefs about smoking, willingness to quit, quit or moderation attempts
- Interests and concerns after diagnosis – what concerned you most (about your health) after diagnosis
- Support in smoking cessation or alcohol moderation – discussion by healthcare professionals, received support, wishes for support
- Internet use – ways of using the internet for health related interests, which websites do your visit frequently and why
- Views on online interventions for cancer survivors – what would make you use it, what would turn you off, views on cancer specific interventions, other recommendations
