# Supplementary material for: Cancer survivors’ views on digital support for smoking cessation and alcohol moderation: a survey and qualitative study
Source: BMC Public Health. 2021 Sep 27;21:1763. doi: 10.1186/s12889-021-11785-7 (PMC8477484; doi:10.1186/s12889-021-11785-7)
Supplement: Supplementary file 3 — Additional file 3. Focus groups topic guide. [file 12889_2021_11785_MOESM3_ESM.docx]

**Appendix 3**

Topic guide for the first two focus groups

- Experiences with online support
- Need for online support programs in cancer survivors
- Tailoring to cancer survivors
- Preferences for social support in alcohol moderation or smoking cessation efforts
- Monitoring of alcohol use or smoking behaviour
- When to address alcohol moderation or smoking cessation
- How to address cancer survivors specifically
- Visual preferences for online support programs

Topic guide for the last two focus groups

- Discussion of themes that emerged in previous two focus groups and interviews
- Specific topics that an (online) support program should cover
- Tone-of-voice in exercises and informative texts
- Discussion of draft versions of online alcohol moderation and smoking cessation programs
- Last tips for developers and researchers
