# Supplementary material for: Cancer survivors’ views on digital support for smoking cessation and alcohol moderation: a survey and qualitative study
Source: BMC Public Health. 2021 Sep 27;21:1763. doi: 10.1186/s12889-021-11785-7 (PMC8477484; doi:10.1186/s12889-021-11785-7)
Supplement: Supplementary file 4 — Additional file 4. Interview topic guide for healthcare professionals and experts. [file 12889_2021_11785_MOESM4_ESM.docx]

**Appendix 4**

Interview topic guide for healthcare professionals

- Approach – description of patients, how do you discuss AM or SC, how do you tailor your approach to the participant, what tools do you usually advice
- Preconditions for success – preconditions for successful treatment, any characteristic of successful quitters/moderators, how do you keep patients motivated
- Online support - what works well in your experience, what are your views on online programs
- Cancer survivors – experiences with cancer survivors, any differences with the general population

Interview topic guide for experts in development and implementation of online support (for cancer survivors)

- Background – work experience, experience with cancer or cancer survivors
- Development – development process of intervention, recruitment, key factors according to participants, recommendations
- Patient participation – ways to involve patient group, barriers and facilitators, recommendations for improvements
- Specific intervention components – how to best incorporate peer support, tailoring and steer away from a patronizing tone of voice
- Implementation – implementation plan for your intervention, target group reach, involved stakeholders
- Need – views on need for (support in) behavioural changes in cancer survivors, how to accommodate those needs
- Cancer survivors – any differences with the general population
