# Supplementary material for: Cancer survivors’ views on digital support for smoking cessation and alcohol moderation: a survey and qualitative study
Source: BMC Public Health. 2021 Sep 27;21:1763. doi: 10.1186/s12889-021-11785-7 (PMC8477484; doi:10.1186/s12889-021-11785-7)
Supplement: Supplementary file 5 — Additional file 5. Table S5. Preferences for support in current users vs former users of alcohol and tobacco. [file 12889_2021_11785_MOESM5_ESM.docx]

**Appendix 5**

Table S5
*Preferences for support in current users vs former users of alcohol and tobacco*

| Support preferences | current  smoker (N=29)  n (%) | former smoker (N=132)  n (%) | all (N=161)  n (%) | current drinker (N=186)  n (%) | former drinker (N=29)  n (%) | all (N=215)  n (%) |
| --- | --- | --- | --- | --- | --- | --- |
|  |  |  |  |  |  |  |
| No support | 11 (37.9) | 47 (35.6) | 58 (36.0) | 88 (47.3) | 12 (41.4) | 100 (46.5) |
| Online information | 4 (13.8) | 13 (9.8) | 17 (10.6) | 20 (10.8) | 5 (17.2) | 25 (11.6) |
| Printed information flyer | 1 (3.4) | 11 (8.3) | 12 (7.5) | 19 (10.2) | 3 (10.3) | 22 (10.2) |
| Free online self-management course | 5 (17.2) | 6 (4.5) | 11 (6.8) | 7 (3.8) | 1 (3.4) | 9 (4.2) |
| Self-help book | 3 (10.3) | 6 (4.5) | 9 (5.6) | 7 (3.8) | 0 (0) | 7 (3.3) |
| Peer support | 6 (20.7) | 8 (6.1) | 14 (8.7) | 6 (3.2) | 1 (3.4) | 7 (3.3) |
| Online support from professional (email or video-call) | 3 (10.3) | 1 (0.8) | 4 (2.5) | 7 (3.8) | 0 (0) | 7 (3.3) |
| Face-to-face support from professional | 9 (31.0) | 14 (10.6) | 23 (14.3) | 10 (5.4) | 4 (13.8) | 14 (6.5) |
| Other | 1 (3.4) | 0 (0) | 1 (0.6) | 11 (5.9) | 0 (0) | 11 (5.1) |
| Not applicable to my situation | 0 (0) | 28 (21.2) | 28 (17.4) | 14 (7.5) | 4 (13.8) | 18 (8.4) |
| missing (none selected) | 2 (6.9) | 17 (12.9) | 19 (11.8) | 25 (13.4) | 3 (10.3) | 28 (13.0) |

*Note.* Some participants are both (former) drinkers and (former) smokers, hence the sample sizes of the different substance users cannot be added.
